# Good Tea

**Published:** 1891-11

**Authors:** 


### GOOD TEA.

After tea has been steeped in boiling water for three minutes over five-sixths of the valuable constituents are extracted. At the end of ten minutes the leaves are almost entirely exhausted. Prolonged infusion gives no additional strength to the liquid, but it does cause the loss by volatilization of the flavoring principles. Hard waters are to be preferred to soft waters in the teapot, as the hard waters dissolve less of the tannin out of the leaves. The bearing of these laboratory results on the art of making a good cup of tea is obvious.—*Standard.*

